# Radioimmunotherapy of Pancreatic Ductal Adenocarcinoma: A Review of the Current Status of Literature

**DOI:** 10.3390/cancers12020481

**Published:** 2020-02-19

**Authors:** Ashleigh Hull, Yanrui Li, Dylan Bartholomeusz, William Hsieh, Barry Allen, Eva Bezak

**Affiliations:** 1 Cancer Research Institute and School of Health Sciences, University of South Australia, Adelaide, SA 5001, Australia; judy.li@unisa.edu.au (Y.L.); william.hsieh@sa.gov.au (W.H.); eva.bezak@unisa.edu.au (E.B.); 2 Department of PET, Nuclear Medicine & Bone Densitometry, Royal Adelaide Hospital, SA Medical Imaging, Adelaide, SA 5000, Australia; dylan.bartholomeusz@sa.gov.au; 3 Adelaide Medical School, The University of Adelaide, Adelaide, SA 5000, Australia; 4 Faculty of Medicine, Western Sydney University, Liverpool, NSW 2170, Australia; bja1940@outlook.com; 5 Department of Physics, The University of Adelaide, Adelaide, SA 5000, Australia

**Keywords:** pancreatic cancer, radioimmunotherapy, radiolabelled antibodies, alpha particles, beta particles, targeted therapy

## Abstract

Pancreatic ductal adenocarcinoma (PDAC) has long been associated with low survival rates. A lack of accurate diagnostic tests and limited treatment options contribute to the poor prognosis of PDAC. Radioimmunotherapy using α- or β-emitting radionuclides has been identified as a potential treatment for PDAC. By harnessing the cytotoxicity of α or β particles, radioimmunotherapy may overcome the anatomic and physiological factors which traditionally make PDAC resistant to most conventional treatments. Appropriate selection of target receptors and the development of selective and cytotoxic radioimmunoconjugates are needed to achieve the desired results of radioimmunotherapy. The aim of this review is to examine the growing preclinical and clinical trial evidence regarding the application of α and β radioimmunotherapy for the treatment of PDAC. A systematic search of MEDLINE^®^ and Scopus databases was performed to identify 34 relevant studies conducted on α or β radioimmunotherapy of PDAC. Preclinical results demonstrated α and β radioimmunotherapy provided effective tumour control. Clinical studies were limited to investigating β radioimmunotherapy only. Phase I and II trials observed disease control rates of 11.2%–57.9%, with synergistic effects noted for combination therapies. Further developments and optimisation of treatment regimens are needed to improve the clinical relevance of α and β radioimmunotherapy in PDAC.

## 1. Introduction

Pancreatic ductal adenocarcinoma (PDAC) is a highly aggressive malignancy with an unmet clinical demand. Despite advances in conventional therapies, the five-year survival rate of PDAC has remained largely unchanged over recent decades and sits at 9.8% in Australia [1]. Surgery remains the only curative treatment option for PDAC. However, its application is limited to patients with locally resectable disease only (approximately 10%–20% of cases) [2]. Post-surgery, hepatic metastasis, local recurrence and peritoneal dissemination are commonly observed and thought to stem from undetectable or micro-metastases [3]. For patients ineligible for surgery, first-line treatment includes the chemotherapy regimen FOLFIRINOX (a combination of fluorouracil, leucovorin, irinotecan, and oxaliplatin) or gemcitabine. External beam radiotherapy may also be delivered in some cases. Despite evidence demonstrating that these treatments can extend patient survival, they are not curative [4]. Several anatomical and physiological factors contribute to the lack of curative treatments for PDAC including the unfavourable location of the pancreatic vasculature, which complicates surgical resections [5]; the cellular heterogeneity of the pancreas, which limits the effectiveness of using a single treatment type only [6,7]; the stromal barrier and high interstitial pressure within the pancreas, which restrict the entry of intravenous substances including chemotherapy agents [8,9]; and the hypoxic nature of PDAC cells, which reduces the efficacy of radiation treatment [10].

**Figure 1:**
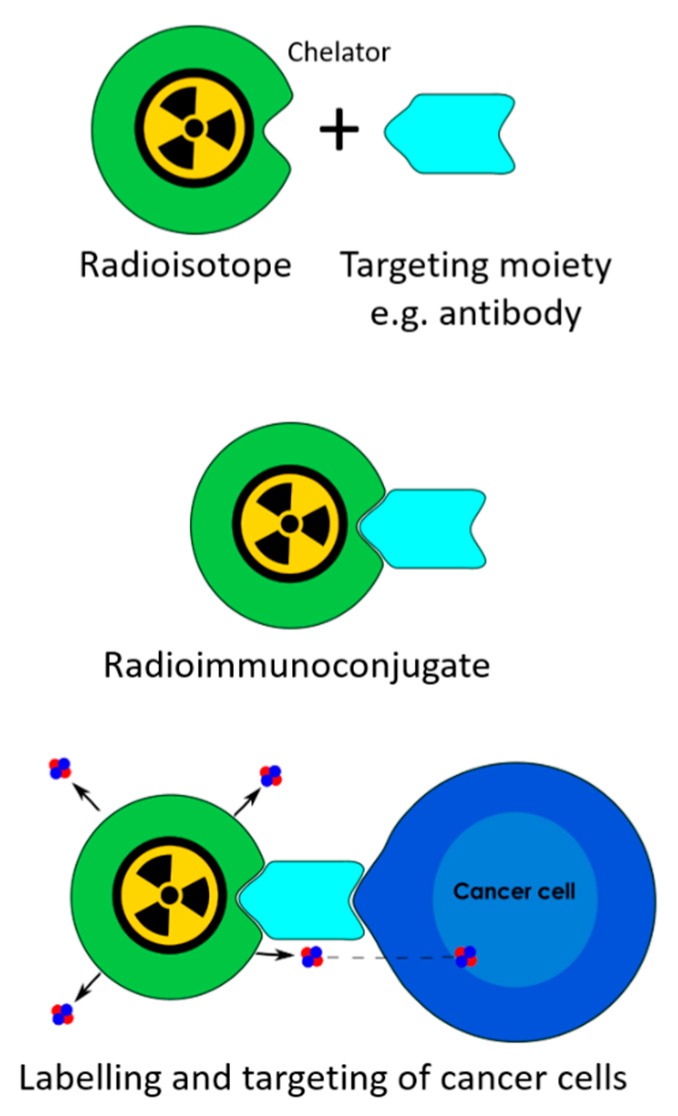
The principles of radioimmunotherapy.

To overcome these barriers, there has been considerable research into new PDAC therapies, such as radioimmunotherapy (RIT). In RIT, α- or β-emitting radionuclides are bound to a monoclonal antibody (mAb) or antibody fragment using a chelator to form a radioimmunoconjugate (Figure 1). These radioimmunoconjugates systemically bind to cancerous cells, delivering a cytotoxic radiation dose directly to the tumours. The level of damage induced in the targeted cells is largely dependent on the radionuclide selected. Whilst β-emitting radionuclides have been more widely used, α-emitting radionuclides are considered superior, particularly for the treatment of micro-metastases [11]. This is primarily due to the high linear energy transfer (LET) of the α particle allowing for dense ionisation damage of the DNA. Consequently, only one direct α particle hit to a cell nucleus is needed to cause complex double-strand DNA breaks and kill an isolated or single cell, even in hypoxic conditions [11]. Several β particle hits are needed to achieve a similar level of cell kill, with a single β particle hit causing repairable damage only [12]. As β particles have low LET, the majority of damage is caused through the production of free radicals, which require a normoxic environment [13,14]. Despite β particles inducing some cell damage, the cytotoxicity of the β particle is insufficient to overcome the hypoxic environment of most solid tumours. However, the longer range and enhanced cross-fire effect of the β particle is advantageous for targeting larger primary tumours. Whilst α particles also induce a cross-fire effect, it is less effective than a β particle, making the α particle more suited for targeting micro-metastases [11,15].

The different physical properties of α- and β-emitting radionuclides provide both α and β RIT with their own advantages and disadvantages for treating PDAC. With growing research into both RIT types, the aim of this review is to evaluate the current preclinical and clinical trial evidence of α and β RIT for the treatment of PDAC. This paper will review the targets and radionuclides investigated in RIT of PDAC, then analyse the studies with respect to study type (in vitro, in vivo and clinical trials) to identify the current status and future directions of research into RIT of PDAC.

## 2. Methods

**Figure A1:**
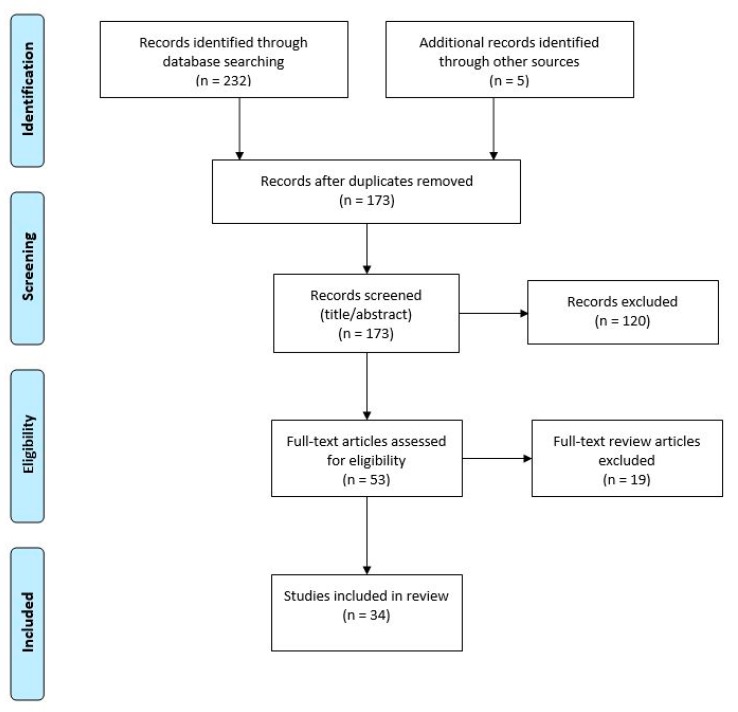
Preferred Reporting Items for Systematic Reviews and Meta-Analyses (PRISMA) flowchart demonstrating the process used to retrieve articles for this review.

The present review was conducted by searching MEDLINE^®^ and Scopus databases using the key terms in Table A1. The search was last conducted in August 2019. Additional studies were also identified by pearling recent articles. The World Health Organisation’s International Clinical Trials Registry and ClinicalTrials.gov were also searched using the key terms “radioimmunotherapy” and “pancreatic cancer” to identify any ongoing or unpublished clinical trials. Duplicate records were removed, and the remaining studies were independently reviewed by two reviewers for inclusion. Studies were excluded if they were reviews, abstract or conference papers, did not investigate RIT or PDAC and were diagnostic in nature. Any disagreements between reviewers were evaluated by a third reviewer. Only full original research papers published since 2000, written in English, and explicitly investigating RIT of PDAC were included in this review. In total, the search and pearling retrieved 238 records—of which, 34 were included in this review following a screening process (Figure A1). Of the retrieved articles, 8 related solely to α RIT and 25 related to β RIT, with one article investigating both approaches.

Whilst the literature was systematically retrieved using the Preferred Reporting Items for Systematic Reviews and Meta-Analyses (PRISMA) guidelines [16], a systematic analysis was not possible due to the high heterogeneity of data across the studies. Instead, identified papers were read in full, with relevant data extracted and tabulated for ease of understanding. The country of origin and year of publication were analysed for both α and β RIT studies as well as the radionuclides and targets used. Finally, all studies were analysed according to the study type—in vitro, in vivo and clinical trials—to allow for a more comprehensive review.

## 3. Results and Discussion

### 3.1. Literature Analysis

**Figure 2:**
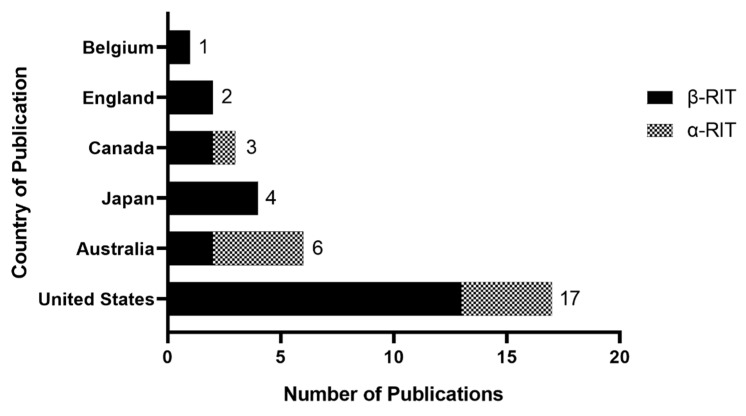
Distribution of publications of radioimmunotherapy (RIT) in pancreatic ductal adenocarcinoma (PDAC) by country of origin.

**Figure 3:**
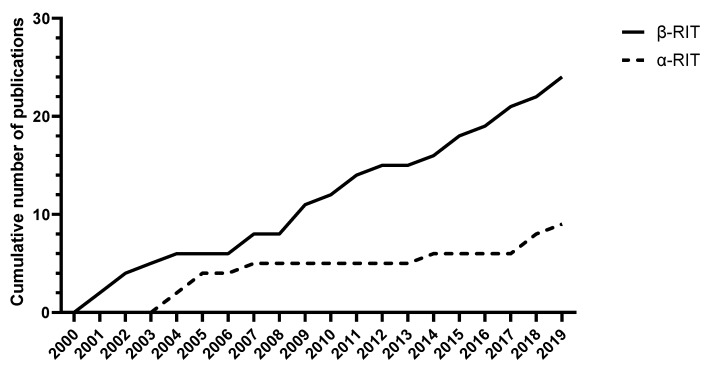
Cumulative distribution of identified publications RIT for PDAC since 2000.

The majority of research into RIT for PDAC has originated from the United States of America (USA), followed by Australia and Japan (Figure 2). Research into α RIT in PDAC has mostly originated from the USA and Australia, with four original research papers published from each country. Since 2000, there has been a steady increase in β RIT publications whilst publications on α RIT in PDAC have been more sporadic (Figure 3). There has been a gradual increase in α RIT publications since 2017, corresponding to increasing interest in targeted α therapies in general.

### 3.2. Radionuclides

Several factors can influence the choice of radionuclide used in RIT, including half-life, range, LET and particle emission. Each of these physical properties has potential to affect the cytotoxicity and tolerability of the radioimmunoconjugate. For example, the half-life of the radionuclide should be sufficient to allow for tumour diffusion but not overly long to cause significant radiation damage to normal tissues [17]. Within the retrieved articles, three β-emitting radionuclides (Yttrium-90 (^90^Y), Lutetium-177 (^177^Lu) and Iodine-131 (^131^I)) and three α-emitting radionuclides (Actinium-225 (^225^Ac), Bismuth-213 (^213^Bi) and Lead-212 (^212^Pb)) have been investigated. The physical properties of the investigated radionuclides all differ as shown in Table 1, where the maximum energy and range in water were calculated using The Stopping and Range of Ions in Matter (SRIM) software (Ziegler 2013).

Due to the difference in range of α and β particles, there is a trade off between homogeneity of tumour dose and radiation-induced normal tissue toxicities. As β particles are capable of traversing over hundreds of PDAC cells, as shown in Table 1, they have an enhanced cross-fire effect compared to α particles, whereby nearby non-targeted cells are also irradiated [18]. This results in a more homogeneous distribution of radiation dose to the tumour. However, it can also increase radiation dose to normal tissues and the likelihood of side effects. The shorter range α particles are capable of only cross-firing to approximately four neighbouring cells, limiting damage to nearby normal cells [11]. The radiation dose delivered to the tumour by the α particles is more heterogenous in nature, with non-irradiated tumour cells continuing to grow, providing one of the major drawbacks of α RIT [15].

The nature of decay of between α and β emitters provides another area of difference. Generally, α emitters undergo a chain of radioactive decay resulting in the formation of several daughter radionuclides. These daughter radionuclides can present additional challenges and need to be appropriately contained when used in RIT to prevent unnecessary damage to normal tissues. Whilst there are less safety implications following β decay, the relative biological effectiveness (RBE) of an α particle is over 100 times greater than that of a β particle. As RBE refers to the amount of radiation needed to induce the same biological effect, this indicates the superior cytotoxicity of the α particle [19]. Given methods can be developed to overcome these safety implications, many would argue that the impressive cytotoxicity of the α particle makes RIT using α-emitting radionuclides the preferred therapeutic approach compared to β RIT [11].

Off-target or indirect radiation effects may also be induced by both α- and β-emitting radionuclides and have potential to affect the safety and efficacy of RIT. The primary off-target effects considered in radiation therapies are the bystander and abscopal effects. The bystander effect refers to the radiation effects induced in non-irradiated cells as a result of cellular signalling from nearby, irradiated cells [20]. The level of bystander effect induced is related to dose, dose rate and LET of the radiation delivered [20]. Most simply, the bystander effect is considered strongest at low LET (e.g., β particles) [21]. The relationship between high LET radiation, such as α particles, radiation dose and bystander effects, is more complex. Boyd et al. [22] suggests there is a U-shaped relationship between radiation dose and the bystander effect when high LET radiation is used. Further research into the bystander effect in RIT is needed to elucidate this finding. A distant bystander effect, known as the abscopal effect, is also yet to be fully evaluated in RIT. The abscopal effect refers to the immune-mediated biological effects observed in distant, non-irradiated cells [20]. Although considered a rare phenomenon, the abscopal effect can cause shrinkage of non-irradiated tumour cells and its presence may also be related to the radiation dose or LET [23]. Despite limited research into the off-target effects of RIT specifically, it remains important to consider their potential to enhance damage to both normal and cancerous cells when developing clinically relevant RIT regimens.

### 3.3. Targets

An ideal RIT target should be selected based on its expression profile on cancerous and normal cells, low blood circulation and high affinity to the intended radioimmunoconjugate. Across the analysed articles, a total of 16 different targets have been investigated. Many of these targets play some role in the progression of cancer and are known to be aberrantly overexpressed in PDAC. Table 2 summarises the expression of these targets (excluding intracellular targets such as single strand DNA and La ribonucleoprotein) in PDAC tissues using immunohistochemical (IHC) staining. For those targets where IHC data is not available, flow cytometry or immunoreactivity data in PDAC cell lines is presented instead. Whilst a RIT target should ideally be overexpressed in cancerous cells, but have minimal to no expression in normal cells, the target expression in normal human tissues is rarely presented. As such, this data is not included in Table 2.

Of the 15 targets in this review, only mucin 1 (MUC1), carbohydrate antigen 19.9 (CA19-9) and centrin 1 (CENT1) have been investigated in both α and β RIT for PDAC. In the healthy pancreas, MUC1 is expressed by both acinar and ductal cells and has a role in cell differentiation and signalling [38]. However, in cancerous cells, MUC1 is aberrantly glycosylated and expressed allowing for targeting of cancer-specific MUC1 epitopes (MUC1-CE) [38]. The exact role of MUC1 in cancer progression is unknown, although it is believed to have anti-adhesion properties which allow cells to evade immunological defences [38].

CA19-9, a tumour-associated antigen, is considered the most accurate serum biomarker for PDAC [39]. As CA19-9 levels are abnormally raised in both malignant and benign pancreatic conditions, the use of this biomarker is predominantly restricted to monitoring tumour growth and treatment success in PDAC patients [40,41]. Whilst CA19-9 does have value as a biomarker, the utility of this antigen as a RIT target remains unclear. Recent research has suggested that CA19-9 is involved in the pathogenesis of PDAC and pancreatitis with increased expression in these diseases [42]. Despite CA19-9 being an appealing therapeutic target, the increased expression in pancreatitis and known free circulation of CA19-9 may lead to unwanted targeting of benign pancreatic tissue and normal tissues throughout the body [43]. The expression of CA19-9 may be less problematic as a diagnostic target where lower energy radionuclides are used [43]. Regardless, further research is warranted to determine the full potential of CA19-9 as a RIT target.

CENT1 is a calcium-binding protein belonging to the cancer testis antigen group. Primarily CENT1 is involved in the mitosis and meiosis of normal cells [29]. Similar to MUC1, the expression of CENT1 is increased by approximately 25-fold in PDAC tissues compared to normal tissues [29]. Whilst the role of CENT1 in PDAC is not yet clear, its expression profile and location in centromeres suggests it may be a valuable RIT target for PDAC [29,44].

### 3.4. In Vitro Studies

**Figure 4:**
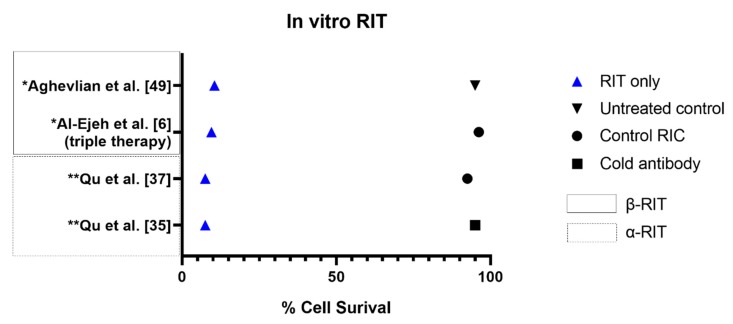
Percentage of PDAC cell survival between RIT and control treatments. * Values estimated from graph. ** Average values used as necessary.

In vitro studies are commonly performed as preliminary experiments to evaluate the feasibility of a therapeutic approach. Whilst in vitro studies are unable to fully assess the biokinetics of an agent and lack replication to the tumour micro-environment, they provide a cost-effective approach to obtaining baseline data without needing animal models [45]. Of the investigated articles, 13 included an in vitro study component. The number of studies investigating α (n = 6) and β RIT (n = 7) were near equal for this component. The results of the in vitro studies are summarised in Table 3. Only studies with quantitative in vitro results which extend beyond flow cytometry analyses for receptor expression are presented in this Table. The cell survival results of selected studies are illustrated in Figure 4.

The objective of the in vitro studies differed between the therapeutic approaches used in the literature. In α RIT, in vitro studies were primarily used to evaluate the inhibition and cytotoxicity of the radioimmunoconjugates whilst in vitro β RIT studies typically assessed cell binding of the radioimmunoconjugate. Given α-emitting radionuclides are expensive and supplies are limited, it is likely to be more cost effective to investigate the cytotoxicity of α immunoconjugates at a cellular level prior to advancing to animal models [52]. As β-emitting radionuclides are more readily available, there is greater flexibility to investigate the inhibitory effects of these conjugates in animal models where the overall conjugate effect can be better investigated. Currently there are no studies comparing α and β RIT at a cellular level.

All α RIT studies demonstrated effective cytotoxicity or inhibitory effects of the radioimmunoconjugate at the in vitro level compared to non-specific isotype control radioimmunoconjugates. The overall dose needed for 37% of cell survival (D0) and the percentage of apoptosis was similar between Qu et al. [37] and Qu et al. [35] despite investigating different targets and ^213^Bi-labelled conjugates. Across the same three PDAC cell lines, Qu et al. [37] demonstrated the D0 ranged from 133 to 185 kBq when targeting the urokinase plasminogen activator/urokinase plasminogen activator receptor (uPA/uPAR). In comparison, Qu et al. [35] showed the D0 varied from 141 to 167 kBq when targeting cancer-specific MUC1 epitopes (MUC1-CE). Similarly, the combined percentage of apoptotic cells at 24 h was 90% when targeting uPA/uPAR and 87% when targeting MUC1-CE with the test radioimmunoconjugates [35,37]. When compared to the control radioimmunoconjugate which produced apoptosis in <15% (MUC1-CE) and 5% (uPA/uPAR) of cells, it can be concluded the therapeutic effect of the test radioimmunoconjugate is due to the overall conjugate, rather than the general radiation effects of ^213^Bi. These studies highlight the potential of α RIT to provide a targeted therapeutic effect.

Whilst in vitro studies are typically limited by not accounting for tumour physiology, replication to the tumour micro-environment can be enhanced by using 3D tumour spheroids. Of the retrieved articles, only Qu et al. [47] and Kasten et al. [46] assessed α RIT in tumour spheroids. Typically, spheroids are more resistant to treatments than adherent 2D cells due to their improved replication to solid tumours [53,54]. The increased radioresistance of the spheroids is evident in Qu et al. [47] where the percentage of apoptotic cells was only 77% at 48 h compared to 92% for the adherent 2D cells used in Qu et al. [35]. Only minor differences were observed in the chelation of the radioimmunoconjugates (^213^Bi-CHX.A”-C595 and ^213^Bi-cDTPA-C595) used in these two studies, further elucidating the radioresistance of solid tumours [35,47]. Kasten et al. [46] found improved inhibitory effects, represented by half maximal inhibitory concentration (IC50) values, in the tumour spheroids compared to the adherent cells (26 ± 17 vs. 41 ± 14). Interestingly, when using the control radioimmunoconjugate, cytotoxicity was greater in the adherent cells than the tumour spheroids (120 ± 12 vs. 180 ± 150) [46]. This is likely due to the general radiation effects of the control radioimmunoconjugate. Unfortunately, tumour spheroids were not used in any of the retrieved β RIT studies.

Only three β RIT studies assessed cytotoxicity at an in vitro level [6,49,50]. Of these studies, Aung et al. [50] investigated the combined efficacy of integrin-targeted β RIT and PI3k/mTOR inhibition. The addition of PI3k/mTOR inhibition to β RIT resulted in a 90.9% reduction in plating efficiency compared to controls at 24 h [50]. For β RIT alone, the plating efficiency was only reduced by 52.5% compared to the controls, suggesting PI3k/mTOR inhibition can sensitise tissues to radiation [50]. Al-Ejeh et al. [6] demonstrated similar tissue sensitisation using a triple combination therapy of epidermal growth factor receptor (EGFR)-targeted β RIT, checkpoint kinase 1 (Chk1) inhibition and gemcitabine. In this study, the clonogenic survival of PANC-1 cells was significantly reduced when using the triple combination therapy compared to any control treatments or the individual therapies [6]. For the remaining β RIT studies, the observed dissociation constants all demonstrated strong affinity between the radioimmunoconjugate and the intended target.

### 3.5. In Vivo Studies

**Figure 5:**
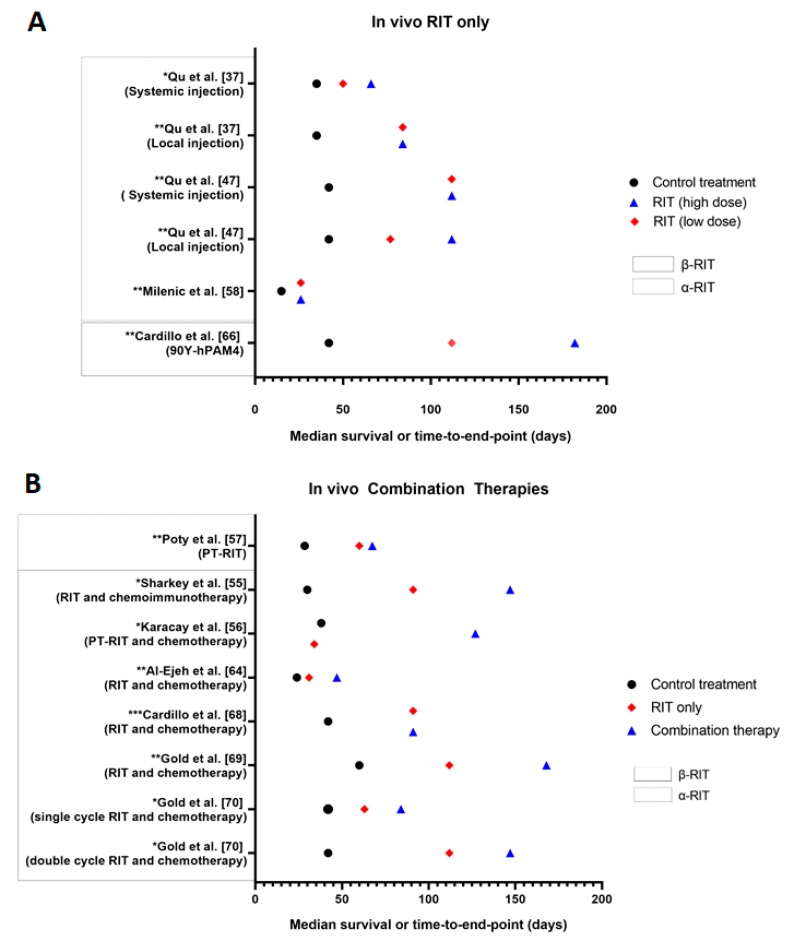
Median survival or time to end point for in vivo studies. A, stand-alone RIT (high and low doses) compared to control treatment (untreated or cold antibody). * *p* ≤ 0.01 and ** *p* ≤ 0.001 for high-dose RIT compared to control treatment. B, RIT in combination with other therapeutic agents (including pre-targeting) for the treatment of PDAC compared to untreated controls. * *p* < 0.05, ** *p* ≤ 0.001, *** *p* ≤ 0.0001 for combined therapy compared to control, except for Sharkey et al. [55] and Karacay et al. [56], where *p*-value compares combination therapy to stand-alone RIT. For Karacay et al. [56], the RIT only data point refers to PT-RIT only. A single representative case is used for studies where multiple experiments were conducted.

In vivo studies are used to evaluate the safety, feasibility, maximum tolerated dose and pharmacokinetics of a RIT approach in mice with human PDAC xenografts [11]. In vivo studies provide a more reliable investigation into the efficacy and tolerability of a therapy than in vitro studies. However, there remains difficulties in extrapolating these findings to clinical in-human trials [45]. In total, 27 of the analysed articles performed in vivo experiments, with eight studies in α RIT and 20 studies in β RIT. One article by Jiao et al. [29] performed a comparison between ^213^Bi and ^177^Lu RIT and thus, is included in both α and β RIT components. The α RIT and β RIT in vivo studies are summarised in Table 4. The median survival results of selected studies are presented in Figure 5.

Many of the in vivo studies demonstrated a significant improvement in survival in the mice who received α or β RIT compared to the control treatments (untreated or unlabelled antibody). Typically, survival improved with higher RIT doses, multiple treatment cycles and the use of a combination therapy approach when delivered within tolerable limits. As an emerging approach, α RIT was primarily assessed as a stand-alone therapy for PDAC, with only Poty et al. [57] evaluating the influence of a pre-targeted approach. In comparison, few in vivo studies focused directly on investigating a single β RIT cycle, with the majority assessing different administration routes, pre-targeting and various combination therapies including the addition of chemotherapy and inhibitors. These variations, along with differences in tumour size, location, observation period and activity administered, limit comparison between the effectiveness of α and β RIT in animals, with both approaches demonstrating value in treating PDAC.

In the only study to compare α and β RIT, Jiao et al. [29] indicate that ^213^Bi α RIT is superior to ^177^Lu β RIT for treating CENT-1 positive PDAC. Jiao et al. [29] found ^213^Bi-69-11 was more effective at suppressing tumour growth compared to ^177^Lu-69-11, with tumour size increasing by only 10-fold in the α RIT group compared to approximately 30-fold in the β RIT group. Additionally, ^213^Bi was found to be as tolerable as ^177^Lu, with transient haemotoxicity the only side effect observed [29]. Whilst further work is needed to harness the full potential of ^213^Bi-69-11, Jiao et al. [29] effectively demonstrates the superiority of α RIT in controlling PDAC tumour growth without compromising on safety and tolerability. Ideally, more studies which directly compare α and β RIT models are needed. However, the variety of radionuclides available for such studies is limited due to the varying chelation requirements of different radionuclides, which can affect the conjugate kinetics and accuracy of the comparison.

Whilst stand-alone RIT had superior therapeutic efficacy compared to most control treatments, RIT in combination with other therapeutic agents further improved tumour control. The most widely investigated combination therapy was gemcitabine and β RIT. However, inhibitors and pre-targeting were also assessed. Between the combined gemcitabine and β RIT studies, Gold et al. [69] demonstrated the greatest improvement in survival in the combination therapy compared to stand-alone RIT groups (24 weeks vs. 16 weeks). However, the same RIT activity of 0.925 MBq (equating to 10% of the pre-determined maximum tolerated dose) was administered to mice in the combination and stand-alone RIT groups [69]. Typically, a lower RIT dose should be administered when it is used in combination with another toxic agent such as gemcitabine due to the synergistic effects and potential for toxicities [71]. As stand-alone RIT has less potential for toxicities, a higher RIT dose can be administered to achieve optimal therapeutic efficacy whilst tolerability is maintained. By prescribing the same RIT activity for both groups, the comparison between the combination and stand-alone RIT treatments is less clinically relevant and may affect translation into clinical studies.

Despite many studies showing positive tumour responses to α and β RIT, there remains concern in the application of these therapies due to the associated toxicities. The primary side effects observed in both in vivo α and β RIT studies were transient weight loss and haemotoxicities. More severe side effects such as disseminated intravascular coagulation, diarrhea, nephrotoxicity and decreasing activity were also observed in both RIT approaches in a limited number of animals [48,56,57,60]. For β RIT, the presence of side effects typically increased with dose or the addition of chemotherapy to the RIT regime. When inhibitors such as PI3k/mTOR and platelet derived growth factor receptor (PDGFR) were administered in combination with RIT, there were no reported side effects [50,65]. As some inhibitors are believed to have radiosensitising properties [72], there is potential to lower the dose of RIT to reduce the level of radiation damage to normal tissues and associated toxicities, without compromising on tumour control. Therefore, the combination of molecular pathway inhibitors and RIT may be valuable for improving the tolerability of RIT treatments.

Nephrotoxicity was observed in some mice receiving α RIT yet not β RIT [57,60]. The effect of α RIT on renal function is likely due to the elimination pathway of the radioimmunoconjugate and recoiling daughter radionuclides. Several free or conjugated α-emitting radionuclides such as ^213^Bi and ^225^Ac-DOTA are excreted via the urinary system [73,74]. Whilst the majority of these agents are rapidly excreted, longer term retention has been observed with some ^213^Bi conjugates [73]. However, the primary safety issue of α-emitting radionuclides lies in the production of recoiling daughter radionuclides following the chain decay of some α emitters, namely ^225^Ac. The recoil energy of these daughter radionuclides is sufficient to overcome the chemical bonds of the conjugate, allowing the radionuclide to dissociate and travel freely within the blood [75]. The most widely considered recoiling daughter product is ^213^Bi which results from the decay of ^225^Ac. With free ^213^Bi known to localise within the kidneys, it is imperative for ^225^Ac conjugates to be internalised with all potentially cytotoxic daughter products to remain contained following α decay [74,75,76]. Clear investigations into methods to limit kidney damage induced by α-emitting radionuclides, either due to recoiling daughter products or general elimination, are needed to improve tolerability of α RIT.

### 3.6. Clinical Trials

**Figure 6:**
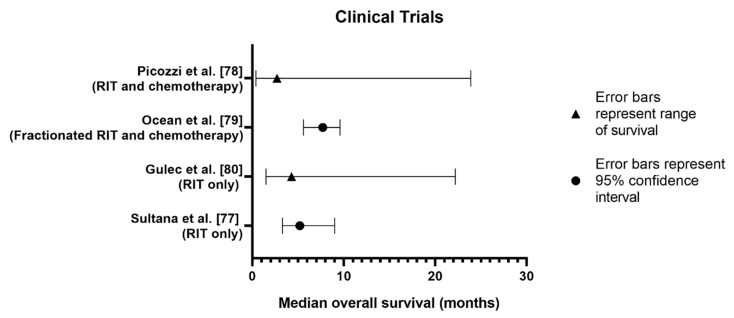
Overall median survival of PDAC patients in β RIT clinical trials.

Clinical trials provide the most reliable data regarding the efficacy and safety of a treatment in humans. In total, there have been five clinical trials investigating β RIT in PDAC. Four of these studies have published results from Phase I and II trials, with the fifth study, a Phase III trial, currently unpublished. The results of these trials are summarised in Table 5 and Figure 6. At the time of searching (August 2019), no clinical trials had been performed investigating α RIT as a treatment for PDAC.

All β RIT trials investigated similar patient populations with stage III or IV PDAC. Sultana et al. [77] had the lowest number of participants (n = 18) whilst Picozzi et al. [78] had the greatest number (n = 58). The greatest variation between trials was the participants’ number of prior treatments. Sultana et al. [77] allowed patients to have had prior treatments although it was not necessary for study enrolment. Ocean et al. [79] recruited previously untreated patients only whilst patients in Picozzi et al. [78] must have received at least two prior chemotherapy regimens. In Gulec et al. [80], stage III patients were required to have had some form of initial therapy whilst stage IV patients needed to have had only one chemotherapy regimen prior to study enrolment. These variations in prior treatments may affect patients’ responses, particularly to combined therapies using gemcitabine, as PDAC is known to develop resistance once exposed to a certain treatment [82]. Additionally, prior treatments such as chemotherapy can cause bone marrow suppression and increase the likelihood of toxicities from subsequent treatments. Picozzi et al. [78] recognised the potential for bone marrow suppression in their cohort who had previous chemotherapy and administered a lower RIT dose to reduce dose-limiting toxicities [78].

Whilst all trials used the Response Evaluation Criteria in Solid Tumours (RECIST) to determine disease control rate, follow-up times varied. Three of the trials determined the disease control rate based on the patients’ best treatment responses between 1 and 3 months post-therapy [77,79,80]. In contrast, Picozzi et al. [78] continued to monitor patients with CT scans every 7–8 weeks and calculated the disease control rate when there was disease progression. The flexible approach of Picozzi et al. [78] allows for a more thorough calculation. However, the longer follow-up time between scans may have falsely extended disease control time.

Most β RIT trials investigated a MUC1-targeted approach using the ^90^Y-hPAM4 conjugate. Gulec et al. [80] was the first study to investigate the efficacy and safety of stand-alone β RIT using ^90^Y-hPAM4. Despite an initial disease control rate of 35% at four weeks post-therapy, all patients had disease progression by 24 weeks [80]. The addition of a gemcitabine regimen improved disease control to 41.4% and 57.9% respectively in Picozzi et al. [78] and Ocean et al. [79]’s trials and further highlights the potential value of combination therapies. Despite positive disease control rates, ^90^Y-hPAM4 RIT with or without gemcitabine, still requires improvements to treat advanced disease and better improve median survival. In Picozzi et al. [78], overall median survival was only 2.7 months. However, this improved to 3.4 months for patients receiving multiple cycles of stand-alone RIT and 7.7 months for patients receiving multiple cycles of combined RIT and gemcitabine [78]. Survival was generally better in patients with a greater Karnofsky performance status and lower serum CA19-9 levels, indicating the therapies are less effective in targeting more advanced disease [78]. Similar conclusions can be drawn from Ocean et al. [79] where overall median survival was 7.7 months. Median survival was reduced to 6.0 months for stage IV patients only whilst stage III patients experienced median survival of 19.6 months. This further demonstrates both the need for earlier detection of PDAC and improved therapeutic measures to target metastatic dissemination observed in stage IV disease. An unpublished Phase III trial [81] investigating ^90^Y-hPAM4 β RIT combined with gemcitabine also failed to improve median survival. At interim analysis, the trial was prematurely terminated due to a lack of overall improvement in survival in the combined therapy group compared to the gemcitabine-only control group [81]. Currently, data from the phase III trial is unpublished preventing further analysis.

Ocean et al. [79] was the only trial to investigate a fractionated RIT approach in combination with gemcitabine. Fractionated RIT can increase the radiosensitivity of the tumour cells as per the 5 Rs of radiobiology: reoxygenation, repair, radiosensitivity, redistribution and repopulation. Fractionation increases oxygenation (↑ radiosensitivity), reduces time for cells to repair damage from previous radiation fractions (↑ radiosensitivity), allows cells to enter different phases of the cell cycle which varies in radiosensitivity and targets cells which repopulate from unkilled tumour cells. Given Ocean et al. [79] had the greatest disease control rate of 57.9% between the trials, fractionation many have value in the delivery of combination RIT, with the practicality of this approach likely to improve at higher RIT doses [83].

Sultana et al. [77] was the only trial to compare therapeutic efficacy between intravenous and intraarterial injections. It was hypothesised that the intraarterial injection of ^131^I-KAb201 would result in greater therapeutic efficacy and reduced toxicity due to its higher potential drug concentration at the tumours [77]. However, overall median survival was 5.2 months, with no significant difference between the administration routes [77]. Regardless, investigations into different administration routes should be more commonly performed for RIT to optimise clinical regimens.

Overall, haematological toxicities such as anaemia, thrombocytopenia, leukopenia and neutropenia were the most commonly reported adverse events across the clinical trials. The high prevalence of cytopenia within these trials is typical of radiation and chemotherapies, given myelosuppression is often the dose-limiting toxicity of these treatments [84]. Other common adverse events included fatigue, nausea, vomiting and a series of gastrointestinal disturbances. Whilst all trials used the relevant National Cancer Institute Common Toxicity Criteria available at the time of study, there were discrepancies in reporting the grades of adverse events which has prevented in-depth comparison between therapy side effects. Gulec et al. [80] reported the highest percentage of patients experiencing serious treatment-related adverse events with 55%. In comparison, approximately 40% of patients experienced serious adverse events in Ocean et al. [79]’s study whilst only 10% of patients had serious treatment-related adverse events in Picozzi et al. [78]. The lower incidence of serious adverse events in Picozzi et al. [78] may be related to the lower RIT dose delivered (6.5 mCi/m^2^) compared to the other trials (6.5–15.0 mCi/m^2^ and 15.0–25.0 mCi/m^2^, respectively for Ocean et al. [79] and Gulec et al. [80]). Sultana et al. [77] reported 31 potentially treatment-related adverse events.

At study enrolment, all patients had either an Eastern Cooperative Oncology Group (ECOG) performance status of ≤1 or a Karnofsky performance status ≥70%, except for one patient in Ocean et al. [79] who had an ECOG of 2 [77,78,79,80]. Unfortunately, no trial presented follow-up performance status or quality of life data. This has prevented evaluations into the potential symptomatic relief provided by RIT.

### 3.7. Limitations of Review

The data presented throughout the retrieved literature was highly heterogeneous in nature. At the in vitro level, differences in study objective, pancreatic cancer cells/tissues and radionuclide used amongst other factors limited our ability to compare the feasibility of α and β RIT for the treatment of PDAC. Similarly, in vivo studies varied in terms of tumour size, location, activity administered and observation period. This prevented in-depth assessments into the impact of these factors on disease control and tolerability, particularly between stand-alone and combination therapies. Finally, whilst several of the clinical trials included in this review investigated ^90^Y-hPAM4, limited reporting regarding the grades of the adverse events and overall efficacy of the treatments weakened the review. Despite systematically retrieving the literature in this review, the variation in the factors described above prevented a systematic analysis of the data, ultimately limiting the comparison between α and β RIT as a treatment for PDAC.

### 3.8. Overall Discussion

RIT is an evolving therapy with potential to improve the outcomes of several low-survival cancers including PDAC. Through the specific targeting of cancerous cells only, RIT can spare normal tissues to provide a high level of tumour control without compromising on patient safety and tolerability. In the current state, RIT is likely to be most valuable as a neoadjuvant or adjuvant therapy to debulk tumour size and reduce the likelihood of disease recurrence in PDAC. The short range and high LET of the α particle suggest α RIT is more suitable to target undetectable or micro-metastases which often lead to disease recurrence [3]. In comparison, β RIT may be useful for debulking the primary tumour given its longer range and improved cross-fire effect.

Whilst RIT alone can suppress tumour growth, RIT in combination with other therapeutic agents is likely to have the most clinical benefit for patients with PDAC. Combination therapies can have synergistic effects to increase stromal permeability and cytotoxicity. Given the cellular heterogeneity of PDAC, combination therapies can better target all cancerous cells by overcoming the intrinsic resistance of some PDAC treatments. With the development of these combination therapies comes a need to establish the optimal treatment strategy due to the higher toxicity associated with combined treatments. This will include determining the most effective administration route, cycle length, dosage and timing of administration to achieve optimal tumour control and tolerability. Whilst the 5 Rs of radiobiology are largely applied to traditional radiation therapy, they may also be applied to RIT to enhance treatment efficacy.

Gemcitabine and β RIT was the most widely investigated combination treatment in this review. Gemcitabine, a chemotherapy agent, acts as a radiosensitiser whilst also providing its own therapeutic effect [71]. At a preclinical level, β RIT and gemcitabine provided superior tumour control compared to stand-alone β RIT, with acceptable side effects. In clinical trials, this combination also demonstrated positive disease control rates of 41.4%–57.9%. However, improvements in survival were marginal, particularly for advanced disease [78,79,81]. The poor translation to clinical trials may be due to the intrinsic limitations of preclinical studies whereby they are unable to fully evaluate the potential of targeting bulky tumours as well as micro-metastases. The inherent toxicities of combining both treatment approaches may have also contributed to the poor survival improvements, as the maximal tolerated doses of both approaches is limited when treatments are used in combination [85].

Advanced PDAC characterised by wide-spread metastatic dissemination may be better controlled by α RIT than β RIT. The current paucity in the literature regarding clinical α RIT studies for PDAC limits comparison. The primary concern regarding α RIT is the potential for nephrotoxicity due to the elimination pathway of the radioimmunoconjugates and localisation of recoiling daughter products [57,60]. The use of nano-carriers, fast tumour uptake of the radioimmunoconjugate and direct tumour administration may be beneficial for reducing the potential toxicity of α RIT [75]. Establishing the kinetics of the radioimmunoconjugate and daughter radionuclides through internalisation experiments at the in vitro level may also assist in translation to clinical studies. To comprehensively evaluate the potential of α RIT for targeting advanced PDAC, clinical studies are urgently needed.

Despite stand-alone α RIT demonstrating considerable therapeutic potential, a combined or pre-targeted approach may be superior if normal tissue toxicities can be appropriately controlled. If α RIT is to follow a similar path to β RIT, gemcitabine is a potential secondary agent for combined therapy. However, the added effect of gemcitabine to an α RIT regimen may be less pronounced than that observed in combined β RIT studies due to the superior cytotoxicity of the α particle. Further challenges will arise in limiting the normal tissue toxicities associated with combining gemcitabine and α RIT given both agents are already considerably cytotoxic. Alternatively, the addition of molecular pathway inhibitors or pre-targeting may improve cytotoxicity without compromising on tolerability. In this review, the side effects observed in pre-targeted α and β RIT or β RIT in combination with inhibitors, were no worse than those observed in RIT alone, whilst tumour control improved. Largely, these findings are due to the mechanisms involved in pre-targeting and inhibition. In pre-targeting, unlabelled antibodies are initially injected to bind with circulating antigens in the blood. The radioimmunoconjugate is then injected and has increased binding to the tumour cells [43]. The process of pre-targeting improves blood clearance and tumour localisation of the radioimmunoconjugate to minimise radiation damage to normal tissues. Pre-targeting has already shown value in reducing the incidence of nephrotoxicity in α RIT due to the fast uptake of the radioimmunoconjugate [57]. On the other hand, some inhibitors act as radiosensitisers to block relevant tumour signalling processes [50]. Inhibitors are less toxic to normal tissues than gemcitabine and may be better suited for combination therapies with α RIT than more toxic agents. Whilst pre-targeting and inhibition has shown value in animal models, clinical trials are needed to fully evaluate the potential of these therapies in a human population.

## 4. Conclusions

Optimising RIT for the treatment of PDAC is an ongoing challenge, particularly for targeting advanced disease. There is a need to improve clinical translation by balancing tumour control with tolerability and improvements in patient survival. Limited clinical studies in both α and β RIT have so far prevented the ability to identify and optimise an effective RIT treatment strategy. RIT will continue to not succeed in the clinic for the treatment of PDAC unless future studies invest in assessing all aspects of the treatment strategy and not just tumour control alone. Further developments and optimisation of therapy combinations are urgently needed to harness the full potential of RIT for PDAC, especially for α RIT.

## Figures and Tables

**Table 1:** Physical characteristics of α- and β-emitting radionuclides used for RIT of PDAC.

	Half-Life	LET in Water (keV/µm)	Maximum Energy (MeV)	Maximum Range in Water (µm)	Number of PANC-1 Cells Traversed *
A emitters					Maximum
^225^Ac	9.92 d	102	8.4	82.2	3.9
^213^Bi	45.6 m	102	8.4	82.2	3.9
^212^Pb	10.6 h	99.4	8.8	88.5	4.2
Β emitters					Average
^90^Y	2.67 d	2.00	2.28	11400	570
^177^Lu	6.65 d	0.28	0.498	1800	125
^131^I	8.02 d	0.20	0.606	3032	202

* Calculated using the average cell diameter of a PANC-1 pancreatic cancer cell line of 20 µm. LET, linear energy transfer.

**Table 2:** Expression of RIT targets in PDAC.

Target	Antibody	Expression in PDAC Cells or Tissues	Measurement Type	References
B7-H3	Anti-B7-H3 (1027)	88.2% (60/68)	IHC	Loos et al. [24]
CA19-9	5B1 CA19-9	77.7% (7/9) 90.7% (39/43)	IHC IHC	Viola-Villegas et al. [25] Shi et al. [26]
CD147	MEM-M6/1 and HIM6 combination	87.2% (34/39)	IHC	Riethdorf et al. [27]
CEA	Anti-CEA	89.2% (223/250)	IHC	Yamaguchi et al. [28]
CENT 1	Anti-CENT 1	50.0% (10/20)	IHC	Jiao et al. [29]
EGFR	Anti-EGFR (H11)	88.5% (92/104)	IHC	Al-Ejeh et al. [6]
Ferritin	Bz-DTPA-AMB8LK	52% (CAPAN-1 cells)	Immunoreactivity	Sabbah et al. [30]
HER 2	Anti-HER 2	59.9% (124/207)	IHC	Harder et al. [31]
Integrin a_v_ß_5_	14C5	100 (4/4)	IHC	Vervoort et al. [32]
Integrin α_6_β_4_	ITGA6B4	55% (range: 13.1–91.0% across four cell lines)	Flow cytometry	Aung et al. [33]
	439-9B	92.0% (104/113)	IHC	Cruz-Monserrate et al. [34]
MUC1	C595 MA5	90.5% (48/53) 100% (43/43)	IHC IHC	Qu et al. [35] Shi et al. [26]
MUC1/MUC5AC	PAM4	79.1% (34/43)	IHC	Shi et al. [26]
PDGFR-β	Anti-PDGFR-β	100% (5/5)	IHC	Hwang et al. [36]
TRFC	Anti-TFRC (Ber-T9)	80.4% (41/51)	IHC	Ryschich et al. [13]
uPA/uPAR	PAI2	87% (26/30)	IHC	Qu et al. [37]

CA19-9: carbohydrate antigen 19.9, CEA: carcinoembryonic antigen, CENT 1: centrin 1, EGFR: epidermal growth factor receptor, HER 2: human epidermal growth factor receptor 2, MUC1: mucin 1, MUC5AC: mucin 5AC, PDGFR- β: platelet-derived growth factor receptor beta, TRFC: transferrin receptor, uPA/uPAR: urokinase plasminogen activator/urokinase plasminogen activator receptor, PAI2: plasminogen activator inhibitor 2, and IHC: immunohistochemistry.

**Table 3:** Summary of the in vitro studies investigating α and β RIT approaches for the treatment of PDAC.

Study/Objective	Target	RIC	Dissociation Constant ± SEM (nmol/L)	Cell Binding (%)	Survival Using RIC	Survival Using Non-Specific Control RIC	Apoptotic Cells Or γ-H2AX Foci (% ± SEM Where Available)
A-RIT Studies							
Kasten et al. [46] Cell binding	B7-H3	^212^Pb-376.96	Adherent cells: 9.0 ± 1.1 CIC: 21.7 ± 0.7	Internalisation: Adherent cells: 44 CIC: 40	Measure: IC50 ± SEM Adherent cells: 41 ± 14 CIC: 26 ± 17	Measure: IC50 ± SEM Adherent cells: 120 ± 12 CIC cells: 180 ± 150	Not investigated
Qu et al. [37] In vitro cytotoxicity	uPA/uPAR	^213^Bi-PAI2	Not investigated	Not investigated	Measure: activity for 37% cell survival (kBq) CFPAC-1: 133 CAPAN-1 185 PANC-1: 170 At 370 kBq: 5%–10% cell survival	Measure: activity for 37% cell survival 2.2–2.7 MBq for all cell lines At 370 kBq: 90%–95% cell survival	Measure: apoptotic cells At 24 h: CFPAC-1: 92 CAPAN-1: 87 PANC-1: 90 Control RIC: <5%
Qu et al. [47] In vitro cytotoxicity	MUC1	^213^Bi-CHX.A”-C595	Not investigated	Not investigated	Not investigated	Not investigated	Measure: apoptotic cells At 48 h: CAPAN: 73 ± 2.6 PANC-1: 78 ± 1.8 CFPAC-1: 81 ± 3.0 Control RIC: <12 ± 3.0
Qu et al. [35] In vitro cytotoxicity	MUC1	^213^Bi-cDTPA-C595	Not investigated	Not investigated	Measure: activity for 37% cell survival (kBq) CAPAN-1: 167 CFPAC-1: 141 PANC-1: 159 At 370 kBq: 5%–10% cell survival	Measure: activity for 37% cell survival (MBq) 2.2–2.6 for all cell lines At 370 kBq: 90–95% cell survival (control RIC) >95% cell survival (conjugated mAb or intact mAb only)	Measure: Combined cell counts 4 h: 11 8 h: 18 12 h: 42 24 h: 87 48 h: 92 72 h: 81 Control RIC: <15% at all time points
B-RIT Studies							
Sugyo et al. [48] Cell binding	CD147	^111^In-059-053 (diagnostic agent to ^90^Y-059-053)	Intact 059-053: 0.35 CHX-A”-DTPA-059-053: 0.99	^111^In-059-053: 51 in BxPC-3 cells	Not investigated	Not investigated	Not investigated
Aghevlian et al. [49] In vitro cytotoxicity	EGFR	^177^Lu-MCP-panitumumab	Not investigated	RIC: 4.16 ± 0.17 RIC with excess mAB: 0.35 ± 0.01 Control RIC with excess mAB: 1.21 ± 0.18	Measure: clonogenic survival of treated cells compared to untreated controls 1.2 MBq RIC: 7.4-fold decrease 0.6 Mbq RIC: 9.0-fold decrease 0.3 MBq RIC: 1.9-fold decrease	Not investigated	Measure: γ-H2AX foci 1.2 MBq RIC: 3.8-fold increase in density vs. untreated controls
Al-Ejeh et al. [6] In vitro cytotoxicity	EGFR	^177^Lu-anti-EGFR	Not investigated	Not investigated	Measure: clonogenic survival Triple combination therapy (RIT, Chk1i and gemcitabine) significantly reduced clonogenic survival vs. untreated controls	Quantitative results not presented	Not investigated at an in vitro level
Sabbah et al. [30] Cell binding	Ferritin	^111^In- and ^90^Y-labelled Bz-DTPA-AMB8LK, Bz-CHX-AU-DTPA-AMB8LK and Bz-DOTA-AMB8LK.	Not investigated	^111^In-DTPA-AMB8LK: 52 ^111^In-CHX-A”-DTPA-AMB8LK: 43 ^111^In-DOTA-AMB8LK: 24	Not investigated	Not investigated	Not investigated
Vervoort et al. [32] Cell binding	Integrin a_v_ß_5_	^125^I-14C5, ^111^In-DOTA- 14C5 and ^111^In-DTPA-14C5	^125^I-14C5: 0.11 ± 0.01 ^111^In-DTPA-14C5: 0.24 ± 0.02 ^111^In-DOTA-14C5: 0.11 ± 0.03 Control RIC: no specific binding	Internalisation at 24 h ^125^I-14C5: 6.93 ± 0.88 ^111^In-DOTA- 14C5: 49.44 ± 0.75 ^111^In-DTPA-14C5: 36.66 ± 1.42	Not investigated	Not investigated	Not investigated
Aung et al. [50] In vitro cytotoxicity	Integrin α_6_β_4_	^90^Y-ITGA6B4	Not investigated	Not investigated	Measure: colony formation at 24 h RIC and BEZ235: 90.9% reduction of PE vs. control RIC only: 52.5% reduction of PE vs. control	Included in previous column	Measure: γ-H2AX positive cells (mean ± SD) at Day 3 RIC and BEZ235: 19.3 ± 7.0 RIC only: 12.7 ± 4.7 BEZ235 only: 4.0 ± 2.2 Control: 2.3 ± 1.2
Sugyo et al. [51] Cell binding	Transferrin	^111^In-labelled TSP-A01, DOTA-TSP-A01, TSP-A02 and DOTA-TSP-A02 (diagnostic agents for ^90^Y-TSP-A01)	^111^In-TSP-A01: 0.22 ^111^In-DOTA-TSP-A01: 0.28 ^111^In-TSP-A02: 0.17 ^111^In-DOTA-TSP-A02: 0.22	Immunoreactive fraction: ^111^In-TSP-A01 and ^111^In-DOTA-TSP-A02: 1.0	Not investigated	Not investigated	Not investigated

RIC: radioimmunoconjugate, SEM: standard error measurement, CIC: cancer initiating cells, IC50: half maximal inhibitory concentration, mAb: monoclonal antibody, MCP: metal chelating polymer, Chk1i: checkpoint kinase 1 inhibitor, and PE: plating efficiency.

**Table 4:** In vivo studies performed using α and β RIT in PDAC.

Study	Target	RIC	Therapies Assessed	Survival	Tumour Growth	Tumour Uptake (% ID/g ± SD)	Side Effects
A-RIT Studies							
Kasten et al. [46]	B7-H3	^212^Pb-376.96	RIT only	Not investigated	Significant inhibition of tumour growth at all RIT dose levels compared to untreated controls	At 24 h: 14.0 ± 2.1 (RIT) 6.5 ± 0.9 (^212^Pb-control)	Transient weight loss
Poty et al. [57]	CA19-9	^225^Ac-5B1	PT-RIT	Median survival (orthotopic tumours): 67.5 days (37 kBq PT-RIT) 60.0 days (37 kBq RIT only) 32 days (18.5 kBq PT-RIT) 46 days (18.5 kBq RIT only) 28.5 days (vehicle-only control)	Not investigated	At 4 h: 4.6 ± 3.3 (PT-RIT) 15.4 ± 3.5 (conventional RIT) At 72 h: 29.6 ± 6.6 (PT-RIT) 31.1 ± 21.4 (conventional RIT)	All RIT groups: Transient weight loss, mild nephrotoxicity, transient haemotoxicity (more severe in conventional RIT group compared to PT-RIT group) Conventional RIT: disseminated intravascular coagulation (2/10)
Jiao et al. [29]	CENT1	^213^Bi-69-11	Comparison of ^213^Bi-69-11 and ^177^Lu-69-11	Not investigated	3.7–7.4 MBq of ^213^Bi-69-11: Significant reduction in tumour growth rate compared to controls	Not investigated	Transient haemotoxicity
Milenic et al. [58]	HER2	^213^Bi-Herceptin	RIT only	Median survival: 15 days (untreated controls)2 2 days (^213^Bi-control) 26 days (18.5 MBq RIT) 28 days (37 MBq RIT) 26 days (74 MBq RIT)	Not investigated in PDAC xenografts	^111^In-Herceptin 24 h: 19.47 ± 3.04 48 h: 31.00 ± 8.92 72 h: 34.00 ± 10.15 120 h: 29.89 ± 3.96 168 h: 15.34 ± 5.14	Increasing weight loss with dose
Bryan et al. [59]	ssDNA and RNA	^213^Bi-chTNT3	RIT compared to gemcitabine and cisplatin	Survival: 100% at day 65 (RIT, cold chTNT3 and untreated) 40% at day 65 (gemcitabine) 0% at day 15 (cisplatin)	Significant reduction in tumour size for RIT and gemcitabine compared to controls	Ratio of sum of pixels in tumour region to sum of pixels in internal organs: 1 h: 0.18 2 h: 0.22 24 h: 0.72 48 h: 0.68	No RIT-related side effects
Qu et al. [37]	uPA/uPAR	^213^Bi-PAI2	Comparing local and systemic RIT injections	Local injection Time to end point: 35 days (cold PAI2) >84 days (≥111 MBq/kg RIT) Systemic injection Time to end point: 35 days (cold PAI2) 50 days (111 MBq/kg RIT) 66 days (222 MBq/kg RIT)	Local injection Tumour growth in: 0/5 tumours (222 MBq/kg RIT) 3/5 tumours (111 MBq/kg RIT) 5/5 tumours (cold PAI2) Systemic injection Tumour growth in: 3/5 tumours (222 MBq/kg RIT) 5/5 tumours (111 MBq/kg RIT) 5/5 tumours (cold PAI2)	Not investigated	Not reported
Song et al. [60]	uPA/uPAR	^213^Bi-PAI2	RIT only	Time to end point: 175 days (470 MBq/kg RIT) 162 days (590 MBq/kg RIT) Did not reach end-point (350 MBq/kg RIT and control groups)	Not investigated	Not investigated	Body weight loss with increasing dose. Decline in renal function.
Qu et al. [47]	MUC1	^213^Bi-C595	Comparing local and systemic RIT injections	Local injection Time to end point: 42 ± 7 days (cold C595) 74 ± 3 days (^213^Bi-control) 77 days (1.85 MBq RIT) >112 days (3.7–7.4 MBq RIT) Systemic injection 42 days (cold C595) 56 days (^213^Bi-control) >112 days (≥ 111 MBq/kg RIT)	Local injection Tumour growth in: 0/5 tumours (3.7–7.4 MBq RIT) 1/5 tumours (1.85 MBq RIT) 5/5 tumours (cold C595 and ^213^Bi-control) Systemic injection Tumour growth in: 0/5 tumours (≥222 MBq/kg RIT) 2/5 tumours (111 MBq/kg RIT) 5/5 tumours (cold C595 and ^213^Bi-control)	Not investigated	Transient weight loss
B-RIT Studies							
Jiao et al. [29]	CENT1	^177^Lu-69-11	Comparison of ^213^Bi-69-11 and ^177^Lu-69-11	Not investigated	^177^Lu-69-11: No significant reduction in tumour growth compared to control treatments	Clear localisation of RIC in tumour at 24 h	Transient haemotoxicity
Houghton et al. [61]	CA19-9	^177^Lu-DOTA-PEG_7_-T_z_	PT-RIT using 5B1-TCO	Not investigated	Tumour doubling time was significantly increased in 44.4 MBq PT-RIT compared to controls and 14.8 MBq PT-RIT. Tumour volume was reduced in 44.4 and 29.6 MBq PT-RIT compared to controls.	At 120 h: 16.8 ± 3.87 (PT-RIT)	No side effects observed
Sharkey et al. [55]	MUC1	^90^Y-hPAM4	Combined RIT and antibody-drug conjugate (ADC) (hRS7–SN-38)	Median time to progression: 4.3 weeks (untreated) 9.75 weeks (ADC only) 13 weeks (2.78 MBq RIT only) >21 weeks (Combined therapy and 4.8 MBq RIT only)	Tumour-free mice at 21 weeks: 0/9 (untreated control) 1/10 (ADC only) 1/10 (2.8 MBq RIT only) 5/10 (4.8 MBq RIT only) 4/10 (Combined therapy using 2.8 MBq RIT) 9/10 (Combined therapy using 4.8MBq RIT)	At 48 h: 48.4 ± 16.4	Transient weight loss
Aung et al. [62]	Integrin $\alpha_{6}\beta_{4}$	^90^Y-ITGA6B4	Single and double RIT cycles	Not calculated—all mice euthanised at day 27	Growth rates significantly reduced in single and double RIT cycles compared to controls	Not investigated	Increasing haemotoxicity with RIT activity
Aung et al. [50]	Integrin $\alpha_{6}\beta_{4}$	^90^Y-ITGA6B4	Combined RIT and PI3K/mTOR inhibitor (BEZ235)	Not investigated	Compared to controls, tumour growth significantly delayed for: 58 days (2.8 MBq RIT only) 23 days (BEZ235 only) Compared to RIT only, tumour growth significantly delayed for 27 days for combined therapy Compared to BEZ235 only, tumour growth significantly delayed for 41 days for combined therapy.	Not investigated	No side effects observed
Aghevlian et al. [63]	EGFR	^177^Lu-panitumumab	RIT only	Not investigated	Not investigated	At 72 h: 6.9 ± 1.3 (^111^In-MCP-panitumumab) 6.6 ± 3.3 (^111^In-DOTA-panitumumab) 1.9 ± 0.3 (^111^In-DOTA-control) 5.4 ± 0.3 (^111^In-MCP-control) In 100-fold excess of panitumumab: 0.02 ± 0.00 (^111^In-MCP-panitumumab) 0.06 ± 0.02 (^111^In-DOTA-panitumumab)	Not investigated
Aghevlian et al. [49]	EGFR	^177^Lu-panitumumab	RIT only	Not investigated	Tumour growth index at 33 days (mean ± SEM): 2.5 ± 0.3 (RIT) 4.0 ± 0.7 (Control RIC) 6.1 ± 1.1 (cold panitumumab) 5.8 ± 0.5 (untreated)	Absorbed tumour dose for 6 Mbq of RIC: 12.33 ± 0.86 Gy	No significant effects over 14 days
Al-Ejeh et al. [6]	EGFR	^177^Lu-anti EGFR	Combined RIT, gemcitabine and Chk1 inhibition (triple therapy)	Not investigated	Tumour growth rate of all triple therapy dose combinations was significantly less than combined gemcitabine and Chk1 inhibition. Complete tumour regression in triple therapy.	Not investigated	Weight loss with high doses of gemcitabine or RIT
Sugyo et al. [48]	CD147	^90^Y-059-053	Combined RIT and gemcitabine	Survival at day 42: 0% (untreated, cold CD147, 0.925 and 1.85 MBq RIT) 0% and 20% (3.7 MBq RIT in two experiments) 40% (Combined therapy)	Significant suppression of tumour growth in 3.7 MBq RIT and combined therapy groups compared to untreated and gemcitabine only groups	^111^In-059-053 30 min: 1.04 ± 0.16 24 h: 9.23 ± 0.67 48 h: 16.13 ± 0.92 96 h: 16.78 ± 2.61 168 h: 14.98 ± 1.63	Weight loss, diarrhea and decreasing activity with multiple cycles
Sabbah et al. [30]	Ferritin	^90^Y and ^111^In-labelled Bz-DTPA-AMB8LK, Bz-CHX-AU-DTPA-AMB8LK and Bz-DOTA-AMB8LK	RIT only—comparing different conjugates	Not investigated	Not investigated	^90^Y-DTPA-AMB8LK: 24 h: 14.0 ± 7.5 48 h: 18.6 ± 1.9 120 h: 16.2 ± 2.9 ^90^Y-DOTA-AMB8LK: 24 h: 14.1 ± 1.2 48 h: 12.9 ± 2.3 120 h: 11.2 ± 4.5	Not investigated
Vervoort et al. [32]	Integrin a_v_ß_5_	^131^I-14C5	RIT only	Not investigated	Not investigated	^131^I-14C5 1 h: 3.63 ± 0.50 24 h: 11.22 ± 3.31 48 h: 12.16 ± 1.03 72 h: 8.45 ± 0.57 168 h: 6.91 ± 1.84	Not investigated
Al-Ejeh et al. [64]	Intracellular La ribonucleoprotein	^90^Y-DOTA-DAB4	Combination RIT, gemcitabine and cisplatin	Median survival 31 days (2.40 MBq RIT only) 47 days (Combined therapy) 24 days (untreated control)	Tumour doubling time (days ± SEM): 4.44 ± 0.02 (control) 5.87 ± 0.04 (RIT only) 4.88 ± 0.01 (chemotherapy only) 8.53 ± 0.02 (combined therapy)	Not investigated in PDAC model	Not investigated in PDAC model
Sugyo et al. [51]	Transferrin	^90^Y-TSP-A01	RIT only	Not investigated	BxPC-3 tumours: 1.85 and 3.7 MBq RIT significantly delayed tumour growth compared to unlabelled A01. No significant difference in tumour volume between 0.74 MBq RIT and unlabelled A01. MIAPaCa-2: Tumour volumes in 1.85 MBq and 3.7 MBq RIT groups were reduced to 20%. Complete resolution of tumours treated with 3.7 MBq RIT by 6 weeks.	Peak ^111^In-TSP-A01 uptake: 37.5 ± 5.3 at 24 h (MIAPaCa-2) 27.0 ± 10.7 at 96 h (BxPC-3)	Transient decrease in body weight
Baranowska-Kortylewicz et al. [65]	PDGFR	^131^I-CC49	Combined RIT and PDGFR inhibitor (imatinib)	Not investigated	Tumour doubling time (days): 12.86 ± 0.19 (RIT only) 26.06 ± 1.47 (combined therapy) 13.03 ± 0.27 (imatinib only) 9.05 ± 0.05 (untreated control)	At 120 h: 6.06 ± 1.76 (RIC only) 9.03 ± 1.59 (RIC and imatinib)	No side effects
Cardillo et al. [66]	MUC1	^131^I-PAM4 and ^90^Y-PAM4	Comparing RICs as stand-alone treatments	Median survival: 6 weeks (untreated) 13 weeks (13 MBq ^131^I RIT) 12 weeks (19 MBq ^131^I RIT) 17.5 weeks (26 MBq ^131^I RIT) 16 weeks (4.8 MBq ^90^Y RIT) >26 weeks (≥6.5 MBq ^90^Y RIT)	Mean size of tumours at nadir (cm^3^): N/A (untreated and ≤19 MBq ^131^I RIT as tumours never regressed) 0.61 ± 0.24 (26 MBq ^131^I RIT at 7 weeks) 0.78 ± 0.61 (4.8 MBq ^90^Y RIT at 6 weeks) 0.33 ± 0.40 (6.5 MBq ^90^Y RIT at 7 weeks) 0.10 ± 0.07 (8.1 MBq ^90^Y RIT at 9 weeks) 0.19 ± 0.13 (9.6 MBq ^90^Y RIT at 10 weeks)	Radiation dose estimates to tumour (cGy): 8559 (26 MBq ^131^I RIT) 8068 (9.6 MBq ^90^Y RIT)	Weight loss
Gold et al. [67]	MUC1	^90^Y-PAM4	Single RIT	Not investigated	Not investigated	96 h: 39.5 ± 16.4	Not investigated
Cardillo et al. [68]	MUC1	^131^I-PAM4	Combined RIT and gemcitabine	Median survival: 6 weeks (3.7 MBq RIT only) 10 weeks (combined therapy using 3.7 MBq RIT) 9 weeks (combined therapy using 3.7 MBq ^131^I control) 13 weeks (7.4 MBq RIT only) 13 weeks (combined therapy using 7.4 MBq RIT) 10 weeks (combined therapy using 7.4 MBq control RIC) 6 weeks (untreated controls) 5 weeks (gemcitabine only)	Normalised tumour growth at week 4: 3.91 ± 2.54 (3.7 MBq RIT only) 1.69 ± 1.26 (combined therapy using 3.7 MBq RIT) 1.45 ± 1.05 (combined therapy using 3.7MBq ^131^I control) 1.58 ± 0.84 (7.4 MBq RIT only) 1.13 ± 0.50 (combined therapy using 7.4 MBq RIT) 1.92 ± 1.02 (combined therapy using 7.4 MBq control RIC) 4.16 ± 0.89 (untreated control) 4.35 ± 1.80 (gemcitabine only)	^131^I-PAM4: 24 h: 12.08 ± 6.85 72 h: 11.08 ± 5.56 168 h: 8.04 ± 6.13 336 h: 4.00 ± 2.80 ^131^I-PAM4 and gemcitabine: 24 h: 12.21 ± 5.73 72 h: 14.29 ± 7.31 168 h: 8.39 ± 6.50 336 h: 2.52 ± 2.30	Weight loss
Gold et al. [69]	MUC1	^90^Y-PAM4	Combined RIT and gemcitabine	Median survival 16 weeks (RIT only) 24 weeks (combined therapy) 11 weeks (combined therapy with ^90^Y control) 8 weeks (^90^Y control only) 10 weeks (gemcitabine only) 8.5 weeks (untreated controls)	Tumour response Week 10: 1/9 PR (RIT only) 1/9 CR, 3/9 PR, 2/9 SD (combined therapy) Disease progression in all other groups Week 26: 4/9 CR (combined therapy)	^111^In-cPAM4: 24 h: 18.65 ± 2.93 96 h: 26.93 ± 11.81 168 h: 18.05 ± 11.02 ^111^In-cPAM4 and gemcitabine: 24 h: 21.79 ± 4.55 96 h: 36.70 ± 9.58 168 h: 25.47 ± 10.35	Weight loss and transient reduction in white blood cell counts
Gold et al. [70]	MUC1	^90^Y-PAM4	Combined RIT and gemcitabine	Median survival 12 weeks (single cycle combined therapy) 9 weeks (single RIT only) 7 weeks (single cycle combined ^90^Y control) 4 weeks (gemcitabine only) 6 weeks (untreated controls) 21 weeks (double cycle combined therapy) 16 weeks (double RIT only) 10 weeks (double cycle combined ^90^Y control)	Tumour response: 1/13 PR, 8/13 SD (single cycle combined therapy) 7/12 SD (single RIT only) 1/8 PR (^90^Y control only) 1/10 CR (single cycle combined ^90^Y control therapy) 1/13 SD (double cycle 1.85 MBq ^90^Y control) 3/13 SD, 1/13 PR (double cycle combined 1.85 MBq ^90^Y control therapy) 3/12 SD, 4/12 PR (double cycle 3.7 MBq RIT) 4/12 SD, 7/12 PR (double cycle combined 3.7 MBq RIT)	Not investigated	Transient weight loss
Karacay et al. [56]	MUC1	PT-RIT: ^90^Y-IMP-288 RIT only: ^90^Y-PAM4	Combined TF10 PT-RIT and gemcitabine	Time to progression 16.3 weeks (9.25 MBq PT-RIT) 5.4 week (untreated controls) >30 weeks (18.5 MBq PT-RIT and 5.55 MBq ^90^Y RIT only) 4.8 weeks (9.25MBq PT-RIT) 18.1 weeks (combined PT-RIT and gemcitabine)	Tumour-free mice at week 19: 8/10 (18.5 MBq PT-RIT) 3/10 (9.25 MBq PT-RIT) 8/9 (5.55 MBq RIT only) 0/10 (untreated controls)	Not investigated	Transient decrease in white blood cell counts, diarrhea (1/11). No nephrotoxicity observed.

RIC: radioimmunoconjugate, %ID/g: percentage of injected dose/gram, SD: standard deviation, PT: pre-targeted, CIT: chemoimmunotherapy, PI3K/mTOR: phosphatidylinositol-3-kinase/mammalian target of rapamycin, MCP: metal chelating polymer, SEM: standard error measurement, Chk1: checkpoint kinase 1, PR: partial response, SD: stable disease, CR: complete resolution.

**Table 5:** Summary of clinical trials performed investigating β RIT in PDAC.

Study	Target	RIC	Study Type	Sample (n)	Patient Group	Median Overall Survival (Months)	Tumour Responses	Disease Control Rate	Adverse Events * (Grade ≥ 3)
Sultana et al. [77]	CEA	^131^I-KAb201	Single RIT comparing IV and IA administration	18	Locally advanced or metastatic PDAC, with at least one tumour site in head of pancreas. KPS ≥ 70, life expectancy < 3 months. Prior treatment allowed but not necessary for inclusion.	5.2 No survival difference between IV and IA administrations	1/18 (5.6%) partial responses 1/18 (5.6%) stable disease 16/18 (88.9%) progression	11.2%	In total, 31 therapy related adverse events were observed. Haemotological toxicity, 18 events; sepsis and vomiting, two events each; alanine aminotransferase, anaemia, anorexia, aspartate aminotransferase, blood alkaline phosphatase, febrile neutropenia, haematemesis, neutrophilia and thrombosis, one event each.
Picozzi et al. [78]	MUC1/MUC5ac	^90^Y-hPAM4	Combination with gemcitabine	58	Metastatic PDAC, ≥ 2 prior chemotherapy regimens with measurable disease by CT. No CNS metastases of single masses ≥10cm. KPS ≥ 70. Adequate haematologic parameters.	2.7 (overall survival for all patients) 7.9 (multiple cycles of combined therapy) 3.4 (multiple cycles of RIT only)	2/29 (6.9%) partial responses (combined therapy group) 10/29 (34.5%) stable disease (combined therapy group) 12/29 (41.4%) stable disease (RIT only group)	41.4%	Thrombocytopenia, 19% of patients (11/58); anaemia, leukopenia and neutropenia, 7% each (4/58), unspecified, 2% (1/58).
Ocean et al. [79]	MUC1	^90^Y-hPAM4	Fractionated RIT combined with gemcitabine	38	Untreated adults with locally advanced or metastatic PDAC. KPS ≥ 70. Life expectancy > 3 months, no CNS tumours or single tumour mass > 10cm. Adequate haematologic parameters.	7.7	6/38 (15.8%) partial response 16/38 (42.1%) stable disease 16/38 (42.1%) disease progression	57.9%	No significant therapy related adverse events occurred.
Gulec et al. [80]	MUC1	^90^Y-hPAM4	Single RIT	20	Stage III or IV PDAC. If stage III, must have progressed after therapy. Stage IV patients must have had no more than one prior chemotherapy regimen. No CNS tumours or single mass > 10cm. KPS ≥ 70 or ECOG ≤1. Adequate haematological parameters.	4.3	At 4 weeks: 3/20 (15.0%) partial response 4/20 (20.0%) stable disease	At 4 weeks: 35.0% Follow up: 0%	Eight therapy related adverse events occurred consisting of seven cytopenia events and a single vomiting event.
ClinicalTrials.gov [81]	MUC1	^90^Y-hPAM4	RIT with gemcitabine	Data not available	Metastatic PDAC, completed at least one prior treatment cycle, progressed following gemcitabine regimen, KPS ≥ 70. No CNS tumours or single mass > 10 cm.	No significant improvements in survival in combined therapy group compared to gemcitabine only group	Data not available	Data not available	Data not available.

* Includes only adverse events considered possibly treatment related. RIC: radioimmunoconjugate, IV: intravenous, IA: intraarterial, KPS: Karnofsky Performance Status, CNS: central nervous system, ECOG: Eastern Cooperative Oncology Group.

## Appendix A

**Table A1:** MEDLINE^®^ Search Strategy used to retrieve articles included within this review.

	Search Terms
1	Pancreatic Neoplasms/
2	Carcinoma, Pancreatic Ductal/
3	(Pancrea * adj3 (neoplasm * or cancer * or carcinoma * or tumo?r *)).mp. [mp = title, abstract, original title, name of substance word, subject heading word, floating sub-heading word, keyword heading word, organism supplementary concept word, protocol supplementary concept word, rare disease supplementary concept word, unique identifier, synonyms]
4	1 or 2 or 3
5	A Particles/tu [Therapeutic Use]
6	Radioimmunotherapy/
7	Β particles/tu [Therapeutic use]
8	(immuno?radio?therap * or radio?immuno?therap * or α therap * or α particle? therap * or α radio?therap * or β therap * or β particle? therap * or β particle? radio?therap * or β radio?therap * or radio?labelled anti?bod * or α immuno?conjugat * or β immuno?conjugat * or radio?immuno?conjugat *).mp. [mp = title, abstract, original title, name of substance word, subject heading word, floating sub-heading word, keyword heading word, organism supplementary concept word, protocol supplementary concept word, rare disease supplementary concept word, unique identifier, synonyms]
9	5 or 6 or 7 or 8
10	4 or 9
11	Limit 10 to (English language and year = “2000–current”)
